# Development and organization of the larval nervous system in *Phoronopsis harmeri*: new insights into phoronid phylogeny

**DOI:** 10.1186/1742-9994-11-3

**Published:** 2014-01-13

**Authors:** Elena N Temereva, Eugeni B Tsitrin

**Affiliations:** 1 Department of Invertebrate Zoology, Biological faculty, Moscow State University, Moscow 119992, Russia; 2 Institute of Developmental Biology, Russian Academy of Sciences, Moscow 117808, Russia

**Keywords:** Phylogeny, Evolution, Lophophorata, Deuterostomia, Protostomia, Larval development, Nervous system, The last common bilaterian ancestor

## Abstract

**Background:**

The organization and development of the nervous system has traditionally been used as an important character for establishing the relationships among large groups of animals. According to this criterion, phoronids were initially regarded as deuterostomian but have more recently been regarded as protostomian. The resolving of this conflict requires detailed information from poorly investigated members of phoronids, such as *Phoronopsis harmeri*.

**Results:**

The serotonin-like immunoreactive part of the *P. harmeri* nervous system changes during larval development. These changes mostly concern the nervous system of the hood and correlate with the appearance of the median and two marginal neurite bundles, the frontal organ, and the sensory field. The apical organ has bilateral symmetry. The tentacular neurite bundle passes under the tentacles, contains several types of perikarya, and gives rise to intertentacular bundles, which branch in the tentacle base and penetrate into adjacent tentacles by two lateroabfrontal bundles. There are two groups of dorsolateral perikarya, which exhibit serotonin-like immunoreactivity, contact the tentacular neurite bundle, and are located near the youngest tentacles. Larvae have a minor nerve ring, which originates from the posterior marginal neurite bundle of the hood, passes above the tentacle base, and gives rise to the mediofrontal neurite bundle in each tentacle. Paired laterofrontal neurite bundles of tentacles form a continuous nerve tract that conducts to the postoral ciliated band.

**Discussion:**

The organization of the nervous system differs among the planktotrophic larvae of phoronid species. These differences may correlate with differences in phoronid biology. Data concerning the innervation of tentacles in different phoronid larvae are conflicting and require careful reinvestigation. The overall organization of the nervous system in phoronid larvae has more in common with the deuterostomian than with the protostomian nervous system. Phoronid larvae demonstrate some “deuterostome-like” features, which are, in fact, have to be ancestral bilaterian characters. Our new results and previous data indicate that phoronids have retained some plesiomorphic features, which were inherited from the last common ancestor of all Bilateria. It follows that phoronids should be extracted from the Trochozoan (=Spiralia) clade and placed at the base of the Lophotrochozoan stem.

## Introduction

The phylum Phoronida is a small group of marine invertebrates with a biphasic life cycle. Adult phoronids are benthic, worm-like animals, and their larvae, which are called actinotrochs, live in plankton. At both larval and adult stages, phoronids have tentacles, which are used to capture food particles and which exhibit some specific peculiarities in filter-feeding mechanisms (for details see [1]). Phoronid larvae live in plankton for several months [2] and then undergo catastrophic metamorphosis [3-5].

The phoronid position among other Bilateria was established by molecular phylogenetic analyses [6,7]. According to these analyses, phoronids are Trochozoan animals, which together with brachiopods form a clade called the Brachiozoa [8,9]. According to recent data [10], phoronids form a group within the brachiopods and are regarded as brachiopods without shells. The protostomian affiliation of phoronids, however, lacks supporting evidence from comparative anatomy and embryology. Moreover, phoronid morphology and embryology have more in common with those of the Deuterostomia than of the Protostomia [11-14]. On the other hand, some recent data revealed that phoronids also have some morphological characters that are not congruent with a strictly deuterostomian interpretation [15-19].

Development and organization of the nervous system has been useful for determining the relationships among different taxa [11,20,21]. The use of features of nervous system development and organization of bilaterian larvae has improved phylogenetic interpretation of some bilaterian groups, including: the relationship between segmented annelids and nonsegmented echiurids and sipunculids, which exhibit metamerism of the nervous system in larvae [21-25]; the protostomian affiliation of brachiopods [26]; and the monophyletic assemblage of Entoprocta + Mollusca [20]. Researchers have several different views regarding the pattern of nervous system organization in phoronid larvae. One view is that phoronid larvae have a deuterostomian-like nervous system [11]. Another view is that the nervous system of phoronid larvae has more in common with the protostomian than with the deuterostomian nervous system [16]. A third view, which is based on the most recent data, is that the organization of the nervous system in phoronid young larvae combines deuterostome- and trochozoan-like features [19]. This disagreement about the organization of the nervous system of phoronid larvae can be partially explained by a lack of breadth in that most studies have been based on *Phoronis* spp. [15,27-31] and less frequently on *Phoronopsis* spp. [18,19]. In addition, most of the investigations listed above used young phoronid larvae, and detailed data about the organization of the nervous system in competent phoronid larvae are nearly absent [16]. At the same time, some new nerve elements appear in phoronid larvae before metamorphosis. Thus, besides having an apical organ, all competent phoronid larvae have a frontal (or pyriform) organ, which apparently plays a main role in larval settlement [4]. A similar organ is known in bryozoan larvae [32], but its homology to the phoronid frontal organ is still uncertain. The collection of novel data concerning nervous system organization in phoronid larvae may reveal common patterns and facilitate comparisons with the nervous system of other main groups of Bilateria with ciliary larvae.

## Results

### Larvae

**Figure 1:**
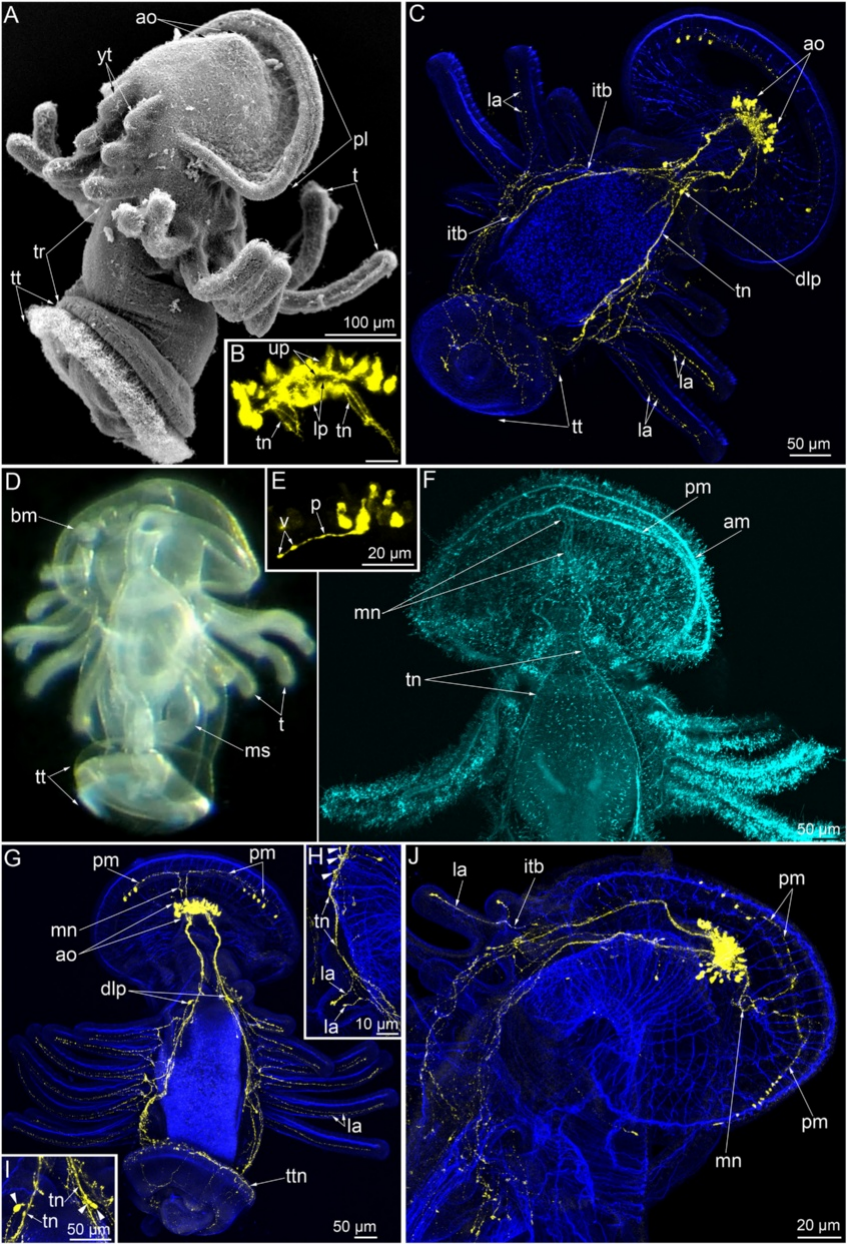
**Serotonin-like immunoreactive nervous system in young larvae of *Phoronopsis harmeri*.** In all images, the apical is at the top, except in C where the apical is to the upper right corner. Z-projections **(B, C, E-J)** of larvae after mono- and double staining for 5-HT (serotonin) (yellow), phalloidin (blue), and alpha-tubulin (cyan). **(A)** Larva with 18 tentacles (SEM); dorsolateral view. **(B)** The apical organ viewed from the dorsal side. **(C)** Dorsal view of larva with 18 tentacles. **(D)** Live larva with 20 tentacles; ventrolateral view. **(E)** Perikaryon with cilium and basal process (p) in the apical organ. **(F)** Dorsal view of the anterior portion of larva with 20 tentacles. **(G)** Dorsal view of larva with 20 tentacles. **(H)** Lateral view of the youngest tentacles: the perikarya of dorsolateral group are indicated by arrowheads. **(I)** Dorsal view of two groups of perikarya (arrowheads), which are located near the youngest tentacles. **(J)** Lateral view of the anterior portion of larva with 20 tentacles. Abbreviations: am – anterior marginal neurite bundle; ao – apical organ; bm – blood mass; dlp – dorsolateral perikarya; itb – intertentacular branch; la – lateroabfrontal neurites in the tentacle; lp – lower portion of the neuropil of the apical organ; mn – median neurite bundle; ms – metasomal sac; pl – preoral lobe; pm – posterior marginal neurite bundle; t – tentacle; tn – tentacular nerve ring; tr – trunk; tt – telotroch; ttn – telotroch nerve ring; v - varicose (node); up – upper portion of the neuropil of the apical organ; yt – youngest tentacles.

**Figure 2:**
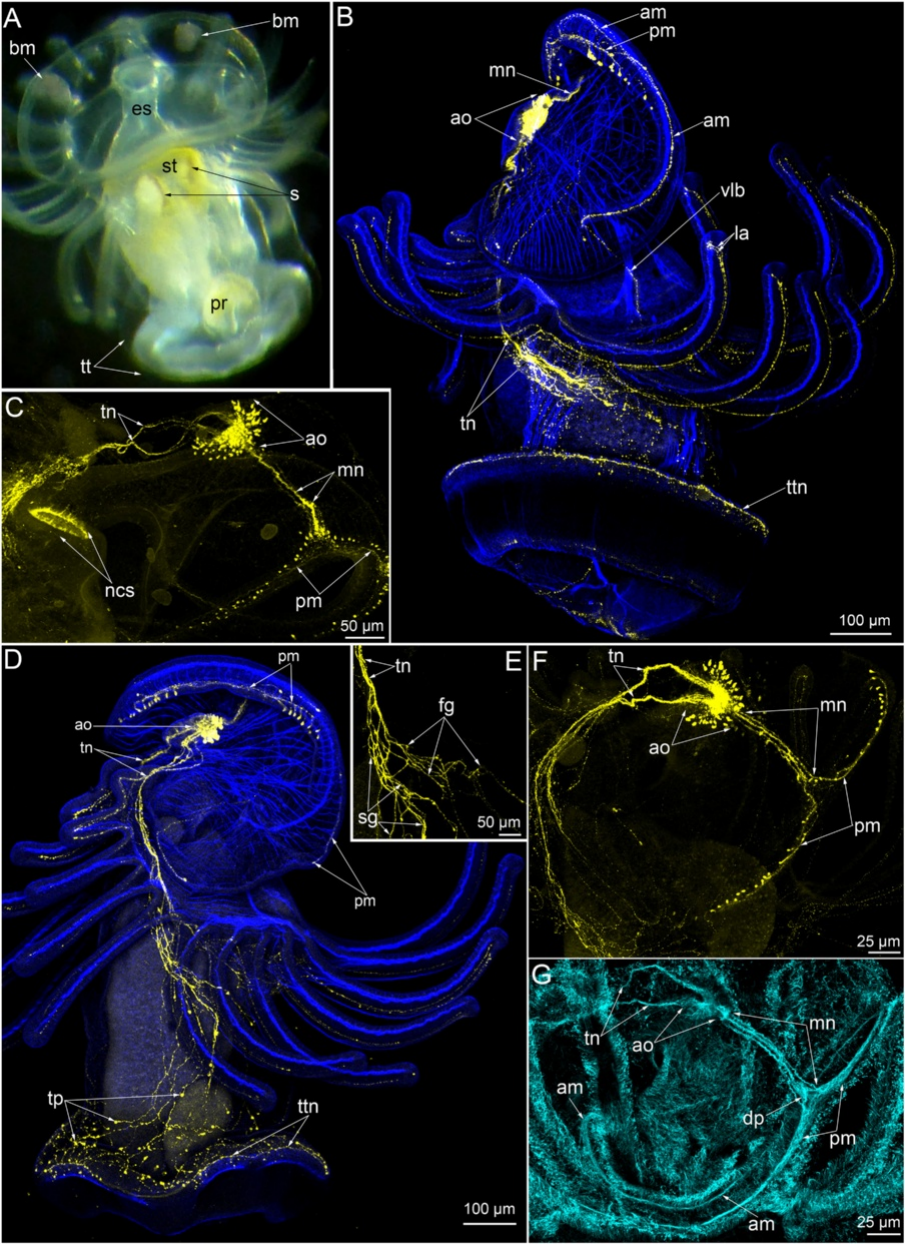
**Serotonin-like immunoreactive nervous system in *Phoronopsis harmeri *larvae with 22 tentacles.** In all images, the apical is at the top, except in A where the apical is to the upper left corner. Z-projections **(B-G)** of larvae after mono- and double staining for 5-HT (serotonin) (yellow), phalloidin (blue), and alpha-tubulin (cyan). **(A)** Ventral view of live larva. **(B)** Whole larva, viewed from the right side. **(C)** Ventrolateral view of the preoral lobe and the esophagus of the larva. **(D)** Dorsolateral view of the whole larva. **(E)** Two groups of neurite bundles, viewed from the right side. **(F, G)** Top view of the preoral lobe; the anterior edge is to the right. Abbreviations: am – anterior marginal neurite bundle; ao – apical organ; bm – blood mass; dp – distal portion of the median neurite bundle; es – esophagus; fg – neurite bundles of the first group; la – lateroabfrontal neurites in the tentacle; mn – median neurite bundle; ncs – neurites of the cardial sphincter; pm – posterior marginal neurite bundle; pr – proctodaeum; s – septum of the stomach; sg – neurite bundles of the second group; st – stomach; t – tentacle; tn – tentacular nerve ring; tp – neurites and perikarya of the trunk; tr – trunk; tt – telotroch; ttn – telotroch nerve ring; vlb – ventrolateral branch.

**Figure 3:**
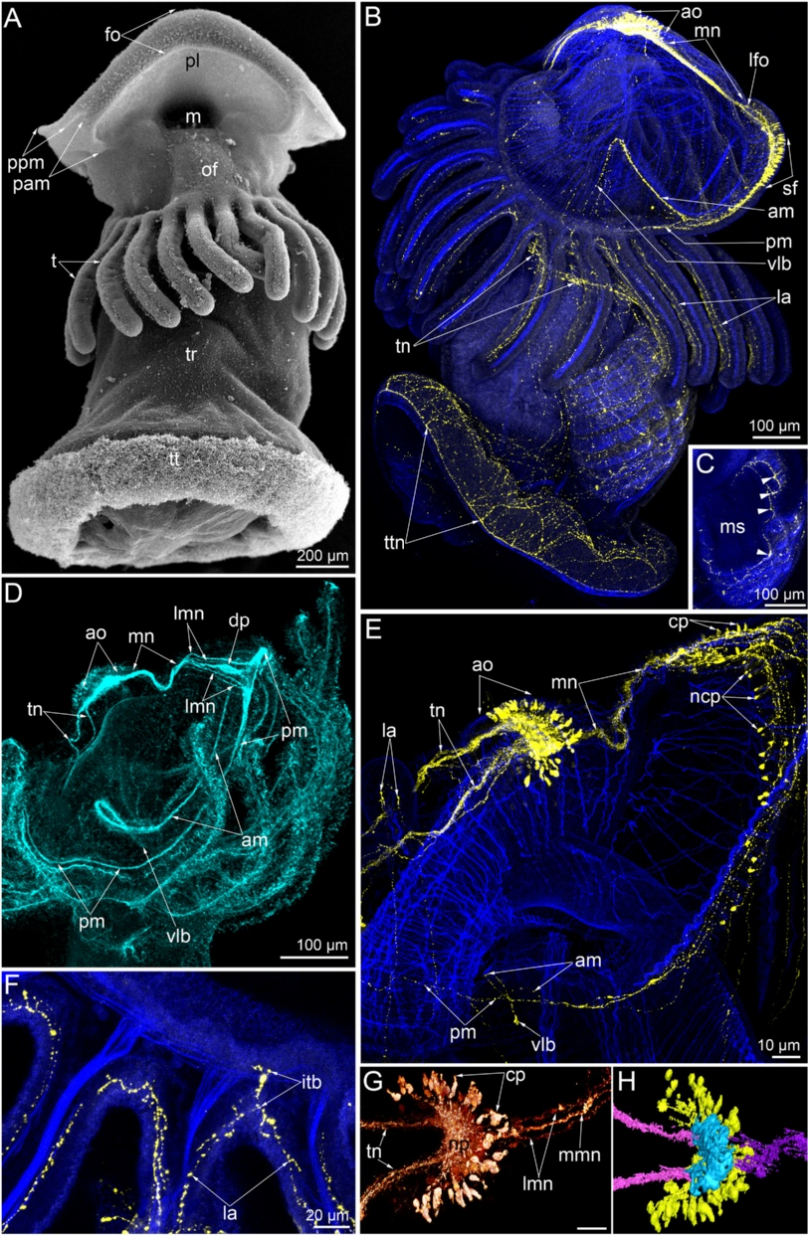
**Serotonin-like immunoreactive nervous system in competent larvae of *Phoronopsis harmeri*.** In all images, the apical is at the top. Z-projections **(B-F)** of larvae after mono- and double staining for 5-HT (serotonin) (yellow), phalloidin (blue), and alpha-tubulin (cyan). **(A)** Competent larva, viewed by SEM from the ventral side. **(B)** Lateral view of competent larva. **(C)** Optical section through the metasomal sac. The perikarya are indicated by arrowheads. **(D)** Anterior part of the larva stained for alpha-tubulin and viewed from the right side. **(E)** Anterior portion of the same larva stained with phalloidin and with 5-HT. **(F)** The base of tentacles. **(G)** Three-dimensional reconstruction of the apical organ viewed from the top. Anterior edge of the preoral lobe is to the right. **(H)** Three-dimensional reconstruction of the apical organ viewed from the bottom. Anterior edge of the preoral lobe is to the right. Color code: yellow – ciliated perikarya, blue – non-ciliated (underlying perikarya), pink – tentacular nerve ring, magenta – median neurite bundle. Abbreviations: am – anterior marginal neurite bundle; ao – apical organ; cp – ciliated perikarya; dp – distal portion of the median neurite bundle; fo – frontal organ; itb – intertentacular branch; la – lateroabfrontal neurites in the tentacle; lfo – “loop” of the frontal organ; lmn – lateral branches of the median neurite bundle; m – mouth; mn – median neurite bundle; ms – metasomal sac; ncp – non-ciliated perikarya; np – neuropil; of – oral field; pam – place of the anterior marginal neurite bundle; pl – preoral lobe; pm – posterior marginal neurite bundle; ppm – place of the posterior marginal neurite bundle; sf – sensory field; t – tentacle; tn – tentacular nerve ring; tr – trunk; tt – telotroch; ttn – telotroch nerve ring; vlb – ventrolateral branch.

Larvae of *P. harmeri* are very abundant in the fall in Vostok Bay, the Sea of Japan, and plankton samples contain *P. harmeri* larvae of different stages. The body of the phoronid larva at different stages is divided into three parts: the preoral lobe (the hood), the collar region with oral field and tentacles, and the trunk. The edge of the hood bears the preoral ciliated band. The postoral ciliated band passes along the laterofrontal sides of the tentacles. The telotroch is located terminally on the trunk and surrounds the anus. The metasomal sac is the invagination of the ventral epidermis under the tentacles. The metasomal sac is located in the trunk coelom and grows with age. Larval stages differ from each other in body size and proportions of body parts; number of tentacles; the presence, number, and color of the blood masses; and the volume of the metasomal sac. The youngest larvae studied here are 600 μm long and have 18 tentacles and lack blood masses (Figure 1A). Larvae with 20 tentacles are 900 μm long and have a tube-like metasomal sac and a pair of dorsolateral blood masses, which are colorless and small in diameter (Figure 1D). Larvae of the next stage are 1200 μm long and have 22 tentacles, a pair of large pale pink blood masses, a looped metasomal sac, and two prominent septa of the stomach (Figure 2A). Competent larvae are 1500 μm long and have 24 tentacles, a pair of large red blood masses on the dorsolateral sides, and 1 to 3 additional small blood masses, which are located in the blastocoel above the tentacles. The metasomal sac of competent larvae occupies most of the trunk coelom. On the ventro-lateral sides, the edge of the preoral lobe is subdivided into two parts: external and internal (Figure 3A). The telotroch of competent larvae is very large and bore numerous long cilia.

### Serotonin-like immunoreactive nervous system: overall anatomy and development

**Figure 4:**
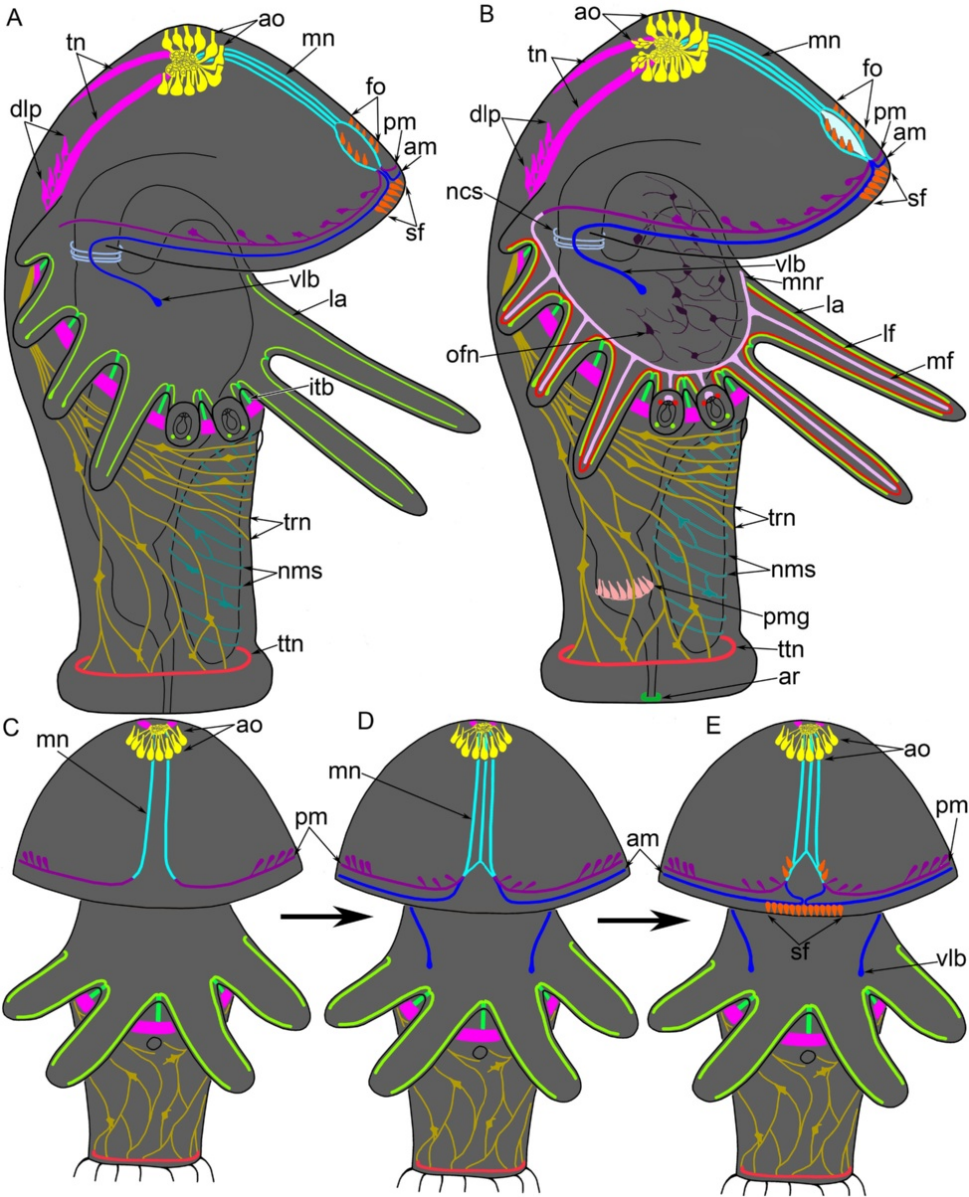
**Schemes of the nervous system organization in *Phoronopsis harmeri *larvae. (A-B)** Competent larvae; the apical organ is at the top; the ventral side is to the right. The number of tentacles has decreased, and the apical organ is simplified. **(A)** Distribution of serotonin-like immunoreactive perikarya and neurites in a competent larva. **(B)** The overall organization of the nervous system in competent larvae, including all nerve elements, which were revealed with all used methods. **(C-E)** The development of the serotonin-like immunoreactive nerve elements of the hood. Larvae are viewed from the ventral side. The organization of larvae is simplified, the number of tentacles decreased. **(C)** Larva with 18-20 tentacles. **(D)** Larva with 22 tentacles. **(E)** Larva with 24 tentacles (competent stage). Abbreviations: am – anterior marginal neurite bundle; ao – apical organ; ar – anal nerve ring; dlp – dorsolateral pericarya; fo – frontal organ; itb – intertentacular branch; la – lateroabfrontal neurites in the tentacle; lf – laterofrontal neurite bundles in the tentacle; mf – mediofrontal neurite bundle of the tentacle; mn – median neurite bundle; mnr – minor nerve ring; ms – metasomal sac; ncs – neurites of the esophagus and cardiac sphincter; nms – neurites and perikarya of the metasomal sac; ofn – neurites and perikarya of the oral field; pm – posterior marginal neurite bundle; pmg – perikarya of the midgut; sf – sensory field; tn – tentacular nerve ring; trn – trunk neurites and perikarya; ttn – telotroch nerve ring; vlb – ventrolateral branch.

Here, we firstly describe the overall anatomy of the serotonin-like immunoreactive nervous system of the larva of *Phoronopsis harmeri* (Figure 4A). We then describe how the serotonin-like immunoreactive nervous system of the preoral lobe changes through the different stages of larval development (Figure 4C-E).

The serotonin-like immunoreactive nervous system consists of several elements. The apical organ and the main tentacular neurite bundle (the main nerve ring, the tentacular nerve ring) are the most prominent elements and were found in all larval stages (Figures 1C, G, 2B, D and 3B). During larval development, the nerve elements of the preoral lobe undergo greater changes than the other elements of the serotonin-like immunoreactive nervous system. Neurites and perikarya of the trunk increase in number with age (Figures 2D and 3B). The telotroch nerve ring becomes more prominent with age (Figures 1G, 2B and 3B). In inner organs, serotonin-like immunoreactive elements occur in the cardial sphincter and are represented by circular neurites, which form a ring between the esophagus and the stomach (Figure 2C). The metasomal sac is also innervated by numerous serotonin-like immunoreactive neurites and perikarya (Figure 3C).

The apical organ occupies the epidermis of the apical plate and consists of perikarya and neurites of different types. In young larvae with 18-20 tentacles, the apical organ is composed of 20-25 perikarya with cilia and 20 other perikarya that do not contact the surface of the apical plate and that do not bear cilia (Figure 1B, C). Flask-shaped perikarya with cilia are arranged in a horseshoe-like pattern along the anterior and lateral edge of the apical plate. The branches of the horseshoe-like structure are directed toward the dorsolateral sides. The flask-shaped perikarya have basal processes, which bear several varicosities (nodes) and pass to the center of the apical plate and form the neuropil (Figure 1E). Perikarya that do not contact the surface of the apical plate are located under the neuropil and are arranged in two lateral groups. As a consequence, the central neuropil is divided into upper and lower portions in transversal optical sections (Figure 1B). The neuropil contacts the basal lamina along the sagittal line but is separated from the basal lamina by underlying perikarya in other areas. The number of ciliated perikaria increases with age and reach 30 in larvae with 22 tentacles (Figure 2C, F) and 37 in competent larvae (Figure 3G). In competent larva, the apical organ includes ciliated flask-shaped perikarya and two groups of underlying perikarya (Figure 3H).

The main tentacular neurite bundle is the most prominent element of the serotonin-like nervous system of *P. harmeri* at all larval stages. In young larvae, the tentacular neurite bundle contains a few perikarya, which with immunocytochemical staining are recognizable on the dorsolateral sides in the base of the youngest tentacles (Figure 1C, G). Here two types of perikarya were revealed by TEM (see below).

The tentacular neurite bundle originates from the lower part of the neuropil of the apical organ and is split into two dorsolateral branches (Figure 1B), which run under the tentacles along the lateral sides of the body and meet on the ventral side. For this reason, the tentacular neurite bundle has been called the “main nerve ring” [16]. Each dorsolateral branch extends from the apical organ as by several neurites, which maintain close contact with each other on the dorsal side and split into numerous thin neurites in the branch points, where the youngest tentacles are located (Figure 1G). At all larval stages studied here, each dorsolateral branch of the main tentacular nerve splits into two groups of neurites (Figure 2E). Neurites of the first group form a net under the base of the tentacles. Individual neurites originate from this net and penetrate into each tentacle. Intertentacular branches are usually present, and these bifurcate in the base of the tentacle and form two branches that extend into adjacent tentacles (Figure 2J). Thus, each tentacle contains two lateroabfrontal serotonin-like immunoreactive neurites, which originate from different intertentacular branches (Figures 1C, H and 3F). In young tentacles, neurites form distal varicosities (Figure 1H and J). Neurites of the second group are more numerous and prominent than neurites of the first group. Neurites of the second group spread along the lateral and ventral sides of the trunk (Figure 2E). These neurites are associated with serotonin-like immunoreactive perikarya, which are scattered in the epidermis of the trunk (Figure 2D).

The telotroch is innervated by a serotonin-like immunoreactive neurite bundle, which is associated with neurites of the trunk and is located in the truncal epidermis adjacent to the epidermis of the telotroch (Figures 1G, 2B and 3B). This neurite bundle forms a circle above the epidermis of the telotroch.

The median neurite bundle develops from the neuropil of the apical organ and passes to the edge of the preoral lobe. In young larvae with 18-20 tentacles, the median neurite bundle consists of two serotonin-like immunoreactive branches (right and left), each of which bends near the preoral lobe edge and passes along it as portion of the posterior marginal neurite bundle (Figures 1G, J and 4C). Each branch ends on the lateral side of the preoral hood, where several serotonin-like immunoreactive perikarya are located and are associated with the neurite bundle (Figure 1G and J). Interestingly, in young larvae with 18-20 tentacles, staining against alpha-tubulin reveals three branches of the median neurite bundle (instead of two serotonin-like immunoreactive branches) and two marginal neurite bundles: a posterior marginal neurite bundle, which has serotonin-like immunoreactivity, and an anterior marginal neurite bundle, which lacks serotonin-like immunoreactivity (Figure 1F).

Among larvae with 22 tentacles, different degrees of complexity are evident in the serotonin-like immunoreactive elements of the preoral lobe. First, the central branch of the median neurite bundle becomes serotonin-like immunoreactive (Figures 2C, F and 4D). This branch originates from the apical organ and passes to the posterior marginal neurite bundle, but does not reach it and is not evident in the most distal end of the hood (Figure 2F). At the same time, staining against alpha-tubulin shows that the distal end of the medial branch forms a bulge, which contacts the posterior marginal neurite bundle (Figure 2G). Serotonin-like immunoreactive pekirarya, which are associated with the posterior marginal neurite bundle, increase in number to 10–13 on each side of the hood (Figure 2C, D and F). In the next step of development, the serotonin-like immunoreactive anterior marginal bundle of the preoral lobe appears (Figure 2B). This neurite bundle passes along the distal edge of the preoral lobe, conducts the annular muscle of the preoral lobe, and passes to the ventrolateral sides of the oral field (Figure 2B).

In competent larvae, the organization of the median neurite bundle is more complicated than in earlier stages. The distal end of the median branch of the median neurite bundle is not recognizable by staining with serotonin. For this reason, the distal end of the median neurite bundle looks like a loop in Z-projections (Figures 3B, E and 4E). The empty space of this loop is occupied by the enlarged end of the median branch, which does not exhibit serotonin-like immunoreactivity but which is revealed by staining with alpha-tubulin (Figure 3D). The distal ends of lateral branches in the median neurite bundle bear numerous flask-shaped cells, which contact the epidermis surface and are probably sensory (Figures 3E and 4E). Perikarya of the posterior marginal neurite bundle increase in number and before metamorphosis form a sensory field along the center of the preoral lobe edge (Figure 2B). This sensory field consists of numerous flask-shaped sensory cells and perikarya, which do not contact the surface of the epidermis (Figure 3B and E). Because the distal edge of the preoral lobe is partially tucked in a vestibulum (Figure 3A), the anterior marginal neurite bundle seems located behind the posterior marginal neurite bundle in Z-projections (Figure 3D). Right and left branches of the anterior marginal neurite bundle continue to the oral field. The posterior marginal neurite bundle is very thick in the center of the preoral lobe but very thin on the lateral and dorsolateral sides (Figure 3B).

In competent larva, the following serotonin-like immunoreactive elements are evident: the apical organ, the tentacular neurite bundle associated with perikarya, paired lateroabfrontal neurite bundles in each tentacle, neurites and perikarya of the trunk, perikarya and neurites in the epidermis of the metasomal sac, the telotroch neurite bundle, median neurite bundles of the preoral lobe, the anterior marginal neurite bundle with ventrolateral protrusions, the posterior marginal nerve associated with serotonin-like immunoreactive sensory cells, and neurites of the cardial sphincter (Figure 4A).

### FMRFamide-like immunoreactive nervous system

**Figure 5:**
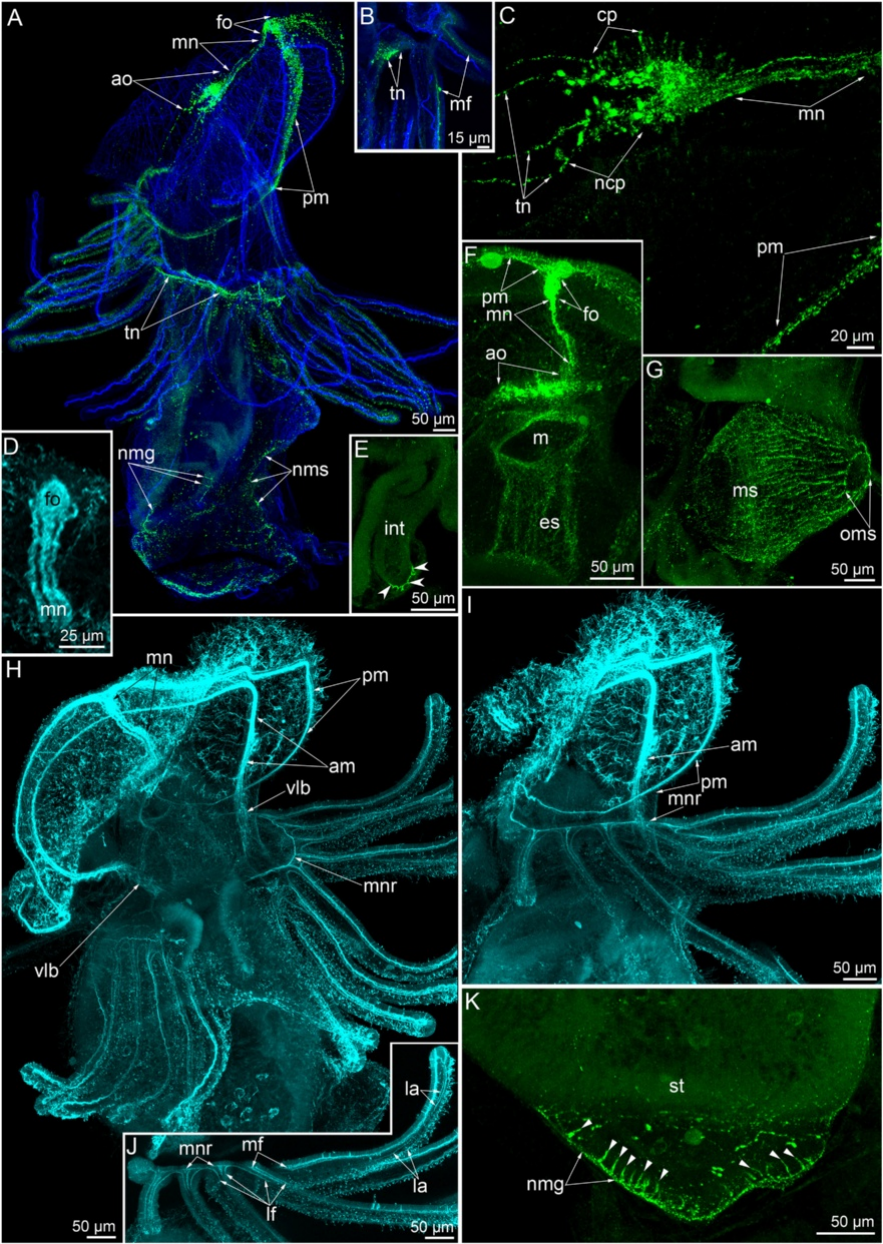
**FMRF-amide-like and alpha-tubulin-like immunoreactive elements in competent larvae of *Phoronopsis harmeri*.** In all images, the apical is at the top, except in H where the apical is to the upper left corner; the ventral side is to the right **(B, C, G, J)**. Z-projections after mono- and double staining with FMRF-amide (green), phalloidin (blue), and alpha-tubulin (cyan). **(A)** Whole larva viewed from the right. **(B)** The part of tentacular neurite bundle (tn) passing under the tentacles and immunoreactive mediofrontal neurite bundle (mf) in the tentacle. **(C)** The apical organ with two types of perikarya; one type contacts the surface of the epidermis (cp) and the other does not (ncp). **(D)** The distal part of the median neurite bundle with the enlarged portion (fo) of the median branch. **(E)** Distal portion of the proctodaeum with the anal nerve ring (open arrowheads). **(F)** Ventral view of the middle portion of the anterior part of the larva with median neurite bundle (mn) and esophageal neurites (es). **(G)** Lateral view of the metasomal sac (ms). **(H)** Ventral view of the anterior portion of the larva stained with alpha-tubulin. **(I)** Lateral view of the anterior portion of the larva stained for alpha-tubulin. **(J)** Several tentacles viewed from frontal and lateral sides. **(K)** The lower part of the stomach (st) and the midgut with immunoreactive perikarya (closed arrowheads) and neurites (nmg). Abbreviations: am – anterior marginal neurite bundle; ao – apical organ; cp – ciliated perikarya; fo – frontal organ (the enlarged portion of the median branch of the median neurite bundle); int – intestine; la – lateroabfrontal neurite bundle in the tentacle; lf – laterofrontal neurite bundle in the tentacle; m – mouth; mn – median neurite bundle; mnr – minor nerve ring; ncp – non-ciliated perikarya; oms – opening of the metasomal sac; pm – posterior marginal neurite bundle; vlb – ventrolateral branch.

In competent larvae, the apical organ is the main element of the FMRFamide-like immunoreactive nervous system (Figure 5A and C). It consists of a huge central neuropil and two groups of perikarya, which are located on the dorso-lateral sides of the apical plate. These perikarya do not bear cilia and do not contact the surface of the epidermis (Figure 5C). Some of the ciliated cells, which are located near the neuropil, exhibit FMRFamide-like immunoreactivity (Figure 5C). Nonciliate perikarya give rise to two dorsolateral branches of the main tentacular neurite bundle. Each branch originates as two neurite bundles (Figure 5C). The FMRFamide-like immunoreactive main tentacular neurite bundle passes under the tentacles and is associated with perikarya, which are located in the epidermis under the tentacles (Figure 5B). In each tentacle, only one FMRFamide-like immunoreactive neurite bundle was found. It passes along frontal side of the tentacle (Figure 5B). The preoral lobe is innervated by median and marginal neurite bundles (Figure 5A and C). The median neurite bundle consists of three branches (Figure 5C). The middle branch is the most prominent and forms a bulge in the distal end near the marginal neurite bundle (Figure 5F). This bulge can be also observed by staining with alpha-tubulin (Figure 5D). The median neurite bundle contacts the posterior marginal neurite bundle, which is associated with numerous FMRFamide-like immunoreactive cells of the epidermis of the preoral lobe (Figure 5A and F). The posterior marginal neurite bundle can be traced along the edge of the preoral lobe, whereas the anterior marginal neurite bundle does not exhibit FMRFamide-like immunoreactivity although it was found in the same larvae by staining with alpha-tubulin (Figure 5H). The posterior marginal neurite bundle continues towards the minor tentacular neurite bundle, which does not exhibit FMRFamide-like immunoreactivity (Figure 5I).

FMRFamide-like immunoreactive neurites and perikarya were found in different organ systems of the competent larvae. Thin longitudinal and circular neurites form a net around the esophagus and the mouth (Figure 5F). Longitudinal neurites were found in the epidermis of the metasomal sac (Figure 5G). Thin circular neurites innervate the metasomal sac opening (Figure 5G). The epithelium of the midgut, contains about 40 FMRFamide-like immunoreactive perikarya (Figure 5K). These are flask-shaped cells that contact the gut lumen; their basal processes form a net around the midgut. The anus is innervated by a circular neurite bundle, which is located in the epithelium on the border between the proctodaeum and the epidermis of the body (Figure 5E).

### alpha-tubulin-like immunoreactive elements

In competent larvae some nerve elements do not exhibit serotonin-like or FMRFamide-like immunoreactivity but can be revealed by staining with alpha-tubulin. The minor nerve ring gives rise to the mediofrontal neurite bundle in each tentacle (Figure 5H-J). Staining with alpha-tubulin facilitates the observation of the laterofrontal neurite bundles in each tentacle (Figure 5J). They contact between the tentacles and form a continuous nervous tract that conducts the postoral ciliated band. Thus, the innervation of tentacles is provided by five longitudinal neurite bundles: one mediofrontal, two laterofrontal, and two lateroabfrontal. The sensory cells of the tentacle also can be found by staining with alpha-tubulin. These cells are located along the laterofrontal sides of each tentacle. The sensory cells are usually grouped in pairs (Figure 5H-J).

### Ultrastructure

Transmission electron microscopy was used to reveal the fine organization of the main nervous system elements. The nature of nerve elements that are 5HT or FMRFamide reactive cannot be recognized, and here we only show the fine organization of the perikarya and neurites, their position with respect to each other, and their location with respect to other organs and tissues.

**Figure 6:**
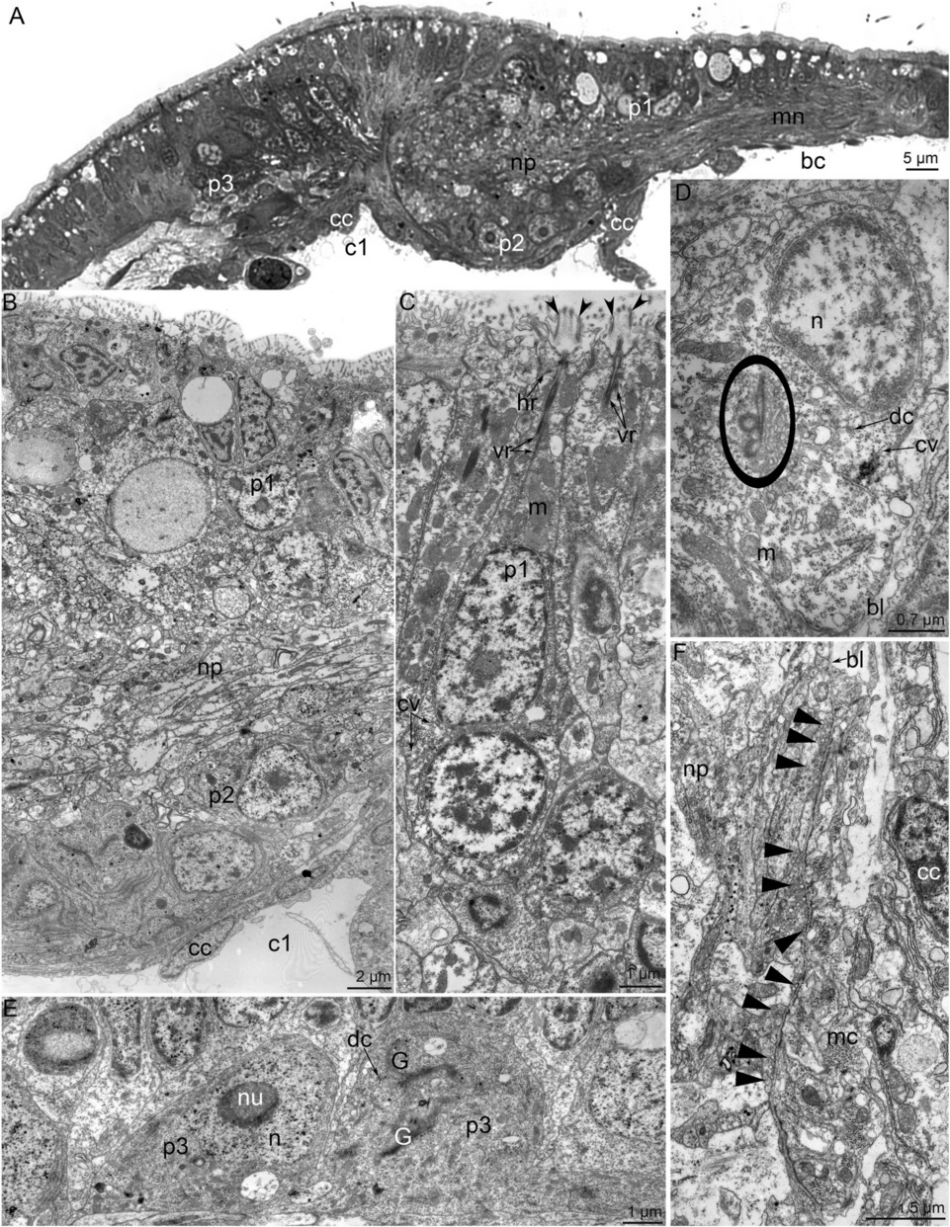
**Details of organization of the apical organ in competent larvae of *Phoronopsis harmeri*. (A)** Semi-thin parasagittal section of the apical organ. The locations of different types of perikarya are shown. The apical side is at the top; the hood edge is to the right; the dorsal side of the larva is to the left. A portion of the median neurite bundle is visible on the right. **(B)** Ultrastructural organization of a portion of the apical organ. Two types of perikarya and the neuropil (np) are visible. **(C)** Ultrastructural details of type 1 perikarya (p1), which have long microvilli (open arrowheads) around the cilium, a root apparatus, large and abundant mitochondria (m), and clear (electron light) synaptic vesicles (cv). **(D)** Ultrastructural details of a type 2 perikaryon (p2), which contains a dense-core (dc), clear (cv) vesicles, and rudiments of a cilium (highlighted by a circle). **(E)** Type 3 perikarya (p3) contain a nucleus (n) with a large nucleolus (nu), a large Golgi apparatus **(G)**, and dense-core vesicles (dc). **(F)** The central portion of the neuropil contacts the basal lamina (bl). Neurites contain numerous synaptic vesicles (closed arrowheads) spread along the thickened membrane. The basal membranes of muscle cells (mc) are also thickened. Abbreviations: bc – blastocoel; bl – basal lamina; c1 – preoral coelom; cc – cells of coelomic lining; cv – clear synaptic vesicles; dc – dense-core vesicles; G – Golgi apparatus; hr – horizontal rootlet; m – mitochondria; mc – muscle cells; mn – median neurite bundle; n – nucleus; np – neuropil; nu – nucleolus; p1 – perikarya of 1 type; p2 – perikarya of 2 type; p3 – perikarya of 3 type; vr – vertical rootlet.

The apical organ of competent *P. harmeri* larvae has a complex histological structure and consists of several types of perikarya (Figure 6A and B). The first type of perikarya is represented by sensory cells. Numerous sensory cells contact the epidermis surface and bear long microvilli, which surround the cilium (Figure 6C). One short horizontal and two long vertical striated rootlets pass from the basal body of the cilium. The apical cytoplasm is filled with mitochondria and vesicles. The large nucleus, which has electron-lucent karyoplasm and bears one or two nucleoli, occupies the central portion of the cell (Figure 6C). Clear synaptic vesicles, 60-70 nm in diameter, are located near the nucleus. The perikarya of the second type do not contact the surface of the epidermis, form two lateral groups under the neuropil, and contact the basal lamina (Figure 6A, B and D). The large nucleus in the second type of perikarya is about 5 μm in diameter and contains a distinct and large nucleolus, which is quite visible even in semi-thin sections. The cytoplasm contains the rudiments of a cilium including a basal body, an accessory centriole, and a short striated rootlet associated with the Golgi apparatus (Figure 6D). Large (90 ± 3 nm) dense-core vesicles and small (40 ± 2 nm) clear (electron light) synaptic vesicles occur in the second type of perikarya. Perikarya of the third type occupy the most dorsal position and form two groups, each of with is located at the beginning of the tentacular nerve ring branches (Figure 6A). This seems very similar to the location of the FMRFamide-like immunoreactive perikarya (Figure 5C). Perikarya of the third type do not contact the surface of epidermis but have the rudiments of a cilium, which is associated with large Golgi apparatuses (Figure 6E). The large nucleus is devoid of peripheral chromatin and contains a large nucleolus. The cytoplasm is grainy and contains many small mitochondria and vesicles, some of which are dense-core synaptic vesicles. The neuropil of the apical organ contacts the basal lamina in areas where the second type of perikarya is absent. Here, neurites contain numerous synaptic vesicles that are spread along the thickened membrane, which contacts the basal lamina. Muscle cells contact the basal lamina on the opposite side and have thickened membranes.

**Figure 7:**
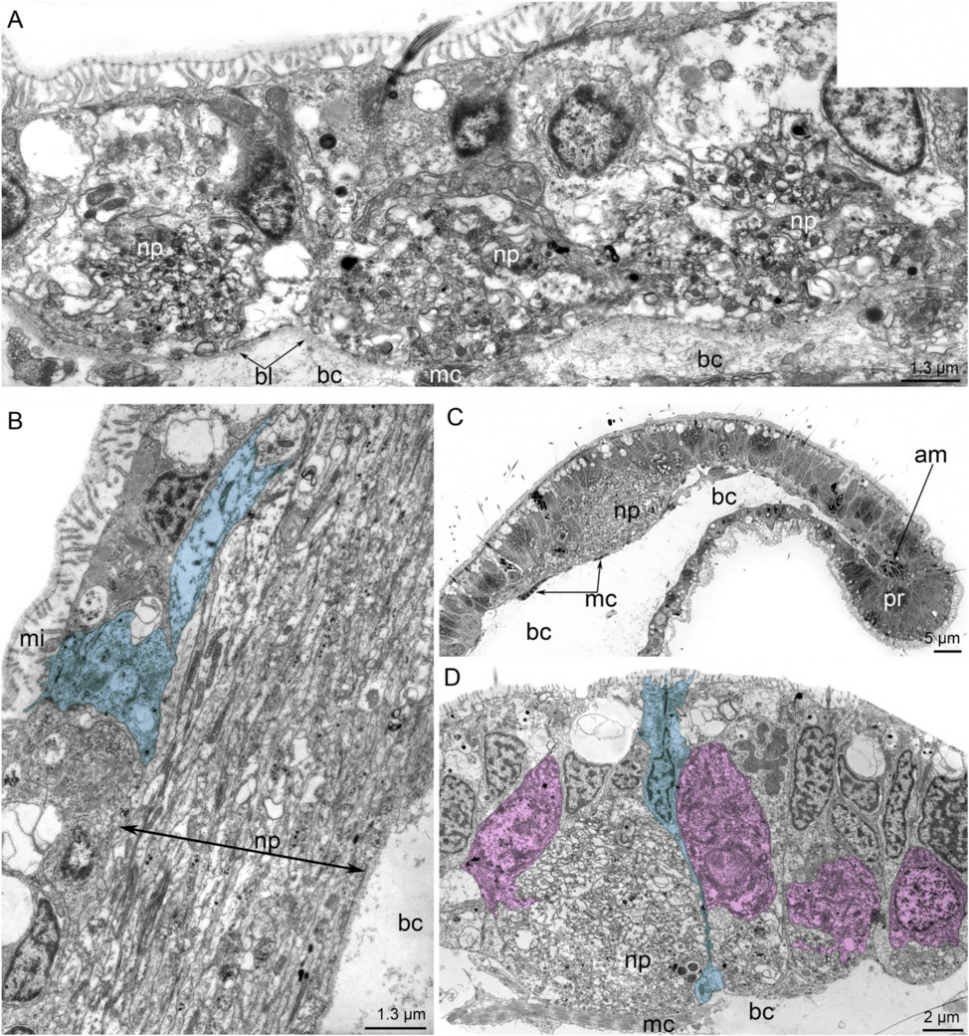
**Details of the organization of the median neurite bundle (A, B) and frontal organ (C, D) in competent larvae of *Phoronopsis harmeri*. (A)** Thin cross section of the exumbrella epidermis of the hood. The median neurite bundle is represented by three bundles of neurites, which intimately contact the basal lamina (bl) and muscle cells (mc). **(B)** Longitudinal section of the median bundle of the median neurite bundle containing a huge neuropil (np) and sensory cell (shown in blue). **(C)** Semi-thin sagittal section of the hood edge. The huge neuropil (np) of the frontal organ is visible. **(D)** The frontal organ neuropil accompanied by sensory (blue) and nonsensory (pink) perikarya. The hood edge is to the right; the dorsal side of larva is to the left. Abbreviations: am – annular muscle of the hood; bc – blastocoel; bl – basal lamina; mc – muscle cells; mi – microvilli; np – neuropil; pr – preoral ciliated band.

**Figure 8:**
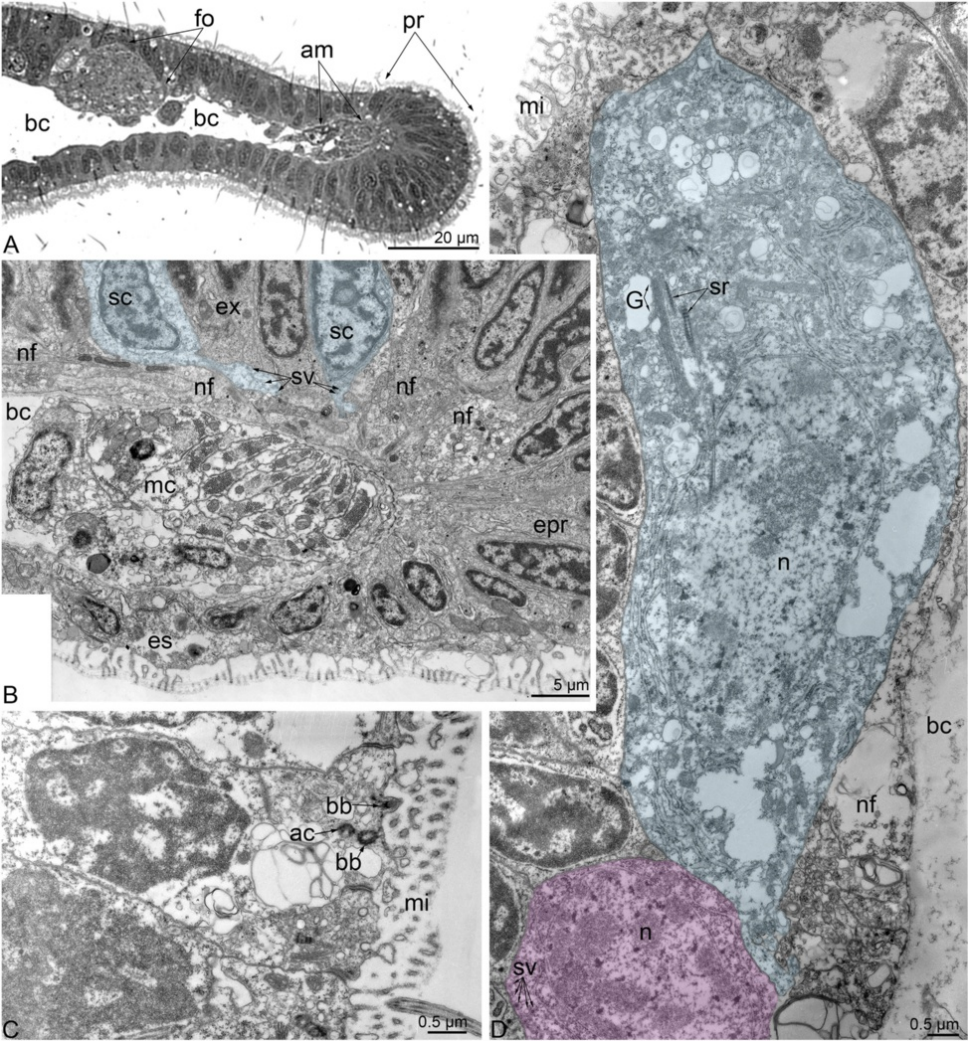
**Ultrastructure of the hood edge in competent larvae of *Phoronopsis harmeri*.** Sagittal semithin **(A)** and thin **(B-D)** sections. **(A)** The hood edge with distal part of the median neurite bundle (fo). **(B)** Fine structure of the marginal neurite bundles. **(C)** Biciliated cells in the epidermis of the hood edge. Two basal bodies (bb) are evident. **(D)** Sensory cell (blue) and perikaryon, which does not contact the surface of the epidermis (pink). Abbreviations: ac – accessory centriole; am – annular muscle of the hood; bc – blastocoel; epr – epidermis of the preoral ciliated band; es – epidermis of the subumbrella; ex – epidermis of the exumbrella; G – Golgi apparatus; mc – muscle cell; mi – microvilli; n – nucleus; nf – nerve fibers; pr – preoral ciliated band; sc – sensory cell; sr – striated rootlet; sv – synaptic vesicles.

The median neurite bundle consists of three interconnected bundles (Figure 7A). Some of the neurites contain large dense-core synaptic vesicles and vesicles with medium-dense content. The sensory cells are scattered along the central bundle of the median neurite bundle. Their upper part contacts the surface of the epidermis, and the basal part forms long projections (Figure 7B). Near the edge of the preoral lobe, the median bundle is greatly enlarged (Figures 7C and 8A) and surrounded by different types of perikarya (Figure 7D).

The sensory field is formed by several types of perikarya that are associated with the posterior marginal neurite bundle. Sensory cells and perikarya, which do not contact the epidermis surface, occur along the middle portion of the posterior marginal neurite bundle (Figure 8B and D). Sensory cells are large, bear a cilium, and have striated rootlets in the cytoplasm (Figure 8D). The basal part of sensory cells forms several processes, which are filled with synaptic vesicles (Figure 8B and D). Perikarya that do not contact the surface of the epidermis are located near the sensory cells and contain dense-core synaptic vesicles (Figure 8D). Some cells of the hood edge are biciliar and contain two basal bodies (Figure 8C).

**Figure 9:**
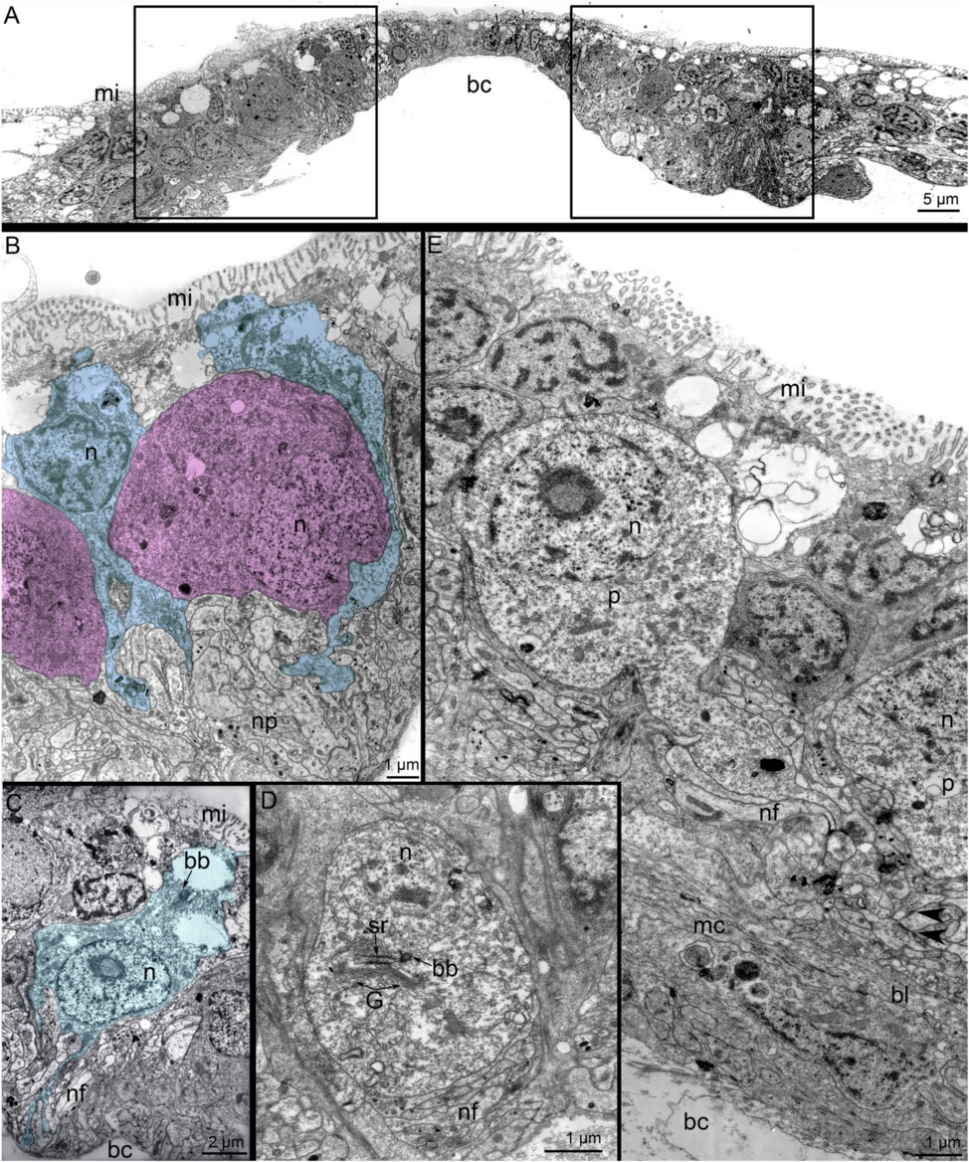
**Ultrastructure of the main tentacular neurite bundle in competent larvae of *Phoronopsis harmeri*.** Thin cross sections. **(A)** A panorama of the two dorsolateral groups of perikarya (indicated by boxes), which are located near the youngest tentacles. The dorsal side of the larva is at the top. **(B)** Ultrastructure of a portion of the dorsolateral group of perikarya, which includes sensory (blue) and nonsensory (pink) cells. **(C)** One of the sensory cells (blue), which are scattered along the tentacular neurite bundle. **(D)** Details of the organization of the nonsensory perikaryon, which is located between neurites of the main tentacular neurite bundle on the lateral side of the larva. **(E)** Two large nonsensory prikarya associated with the tentacular neurite bundle. The synaptic contacts are indicated by arrowheads. Abbreviations: bb – basal body; bc – blastocoel; bl – basal lamina; G – Golgi apparatus; mc – muscle cell; mi – microvilli; n – nucleus; nf – nerve fibers; p – perikaryon; sr - striated rootlet.

**Figure 10:**
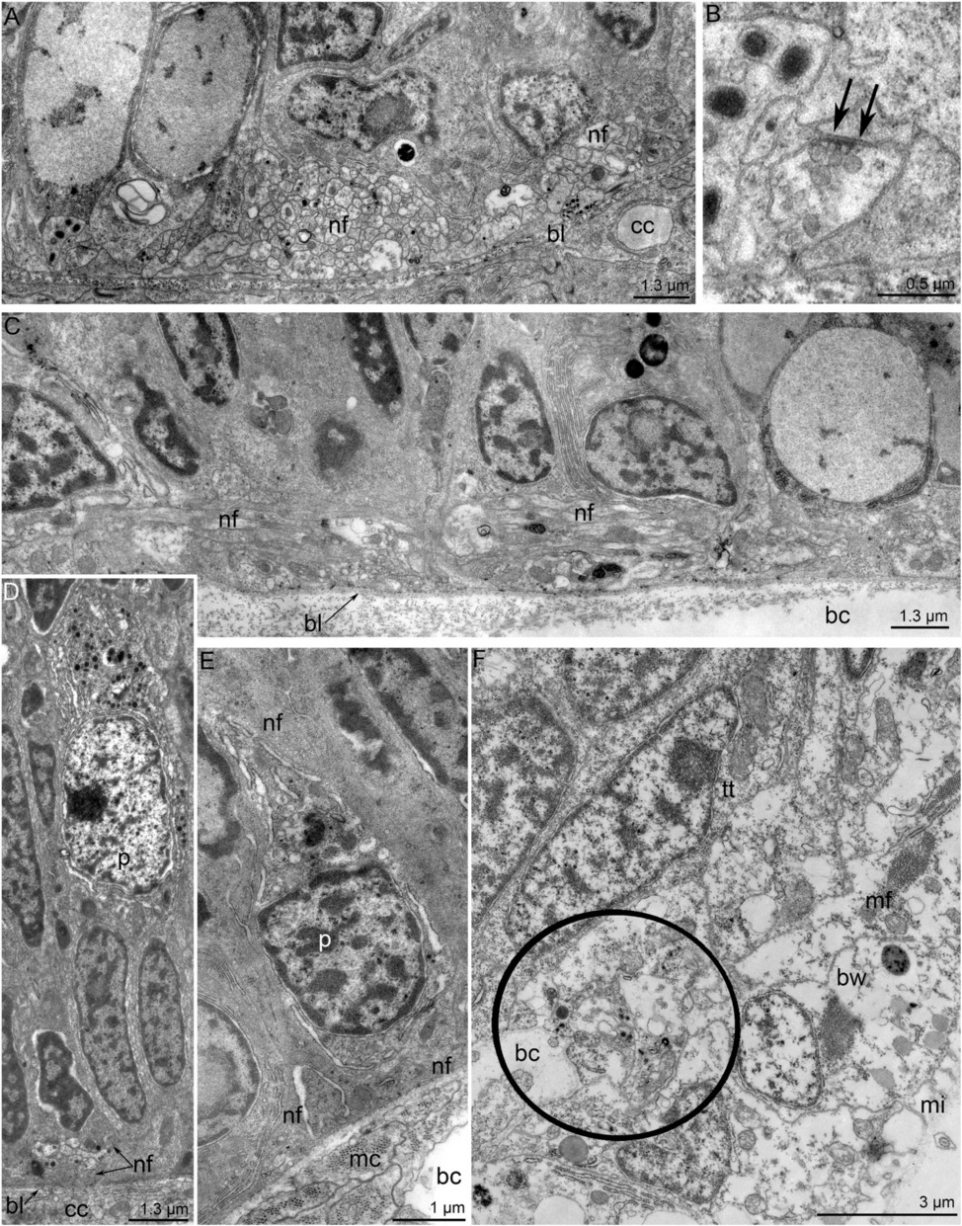
**Ultrastructure of some nerve elements in competent larvae of *Phoronopsis harmeri*.** Thin sagittal sections. **(A)** The most ventral portion of the main tentacular neurite bundle, which consists of several bundles of nerve fibers (nf). **(B)** Synaptic contact (arrows) between neurites of the main tentacular neurite bundle. **(C)** A part of the minor tentacular neurite bundle, which gives rise to the mediofrontal neurite bundle that extends into a tentacle. **(D)** Neurosecretory cell and a bundle of neurites (nf) in the midgut epithelium. **(E)** Perikaryon (p) and nerve fibers (nf) in the epidermis of the oral field. **(F)** Neurite bundle (indicated by a circle) of the telotroch nerve ring, which passes between the epidermis of the body wall (bw) and the epidermis of the telotroch (tt). Abbreviations: bc – blastocoel; bl – basal lamina; bw – epidermis of body wall; mc – muscle cell; mf – miofilaments; mi – microvilli; n – nucleus; nf – nervous fibers; p – perikaryon; tt – epidermis of telotroch.

Dorsolateral perikarya form two large groups in the base of the youngest tentacles (Figure 9A). Each group contains numerous perikarya of two main types: sensory cells and cells that do not contact the surface of the epidermis. The nucleus of sensory cells is small and contains a lot of peripheral chromatin. Sensory cells form several basal processes, which contain synaptic vesicles and surround the non-sensory perikarya (Figure 9B). The perikarya of the second type have large nuclei, which lack peripheral chromatin, and numerous small mitochondria and synaptic vesicles (Figure 9B). Both types of perikarya are scattered along the tentacular neurite bundle but do not form large aggregations (Figure 9C-E). Projections of the first type of neurons contact the basal lamina (Figure 9C). Each non-sensory perikaryon bears a large nucleus with a nucleolus (Figure 9E) and contains the basal body with striated rootlet and Golgi apparatus (Figure 9D). In sagittal sections of the larvae, the tentacular neurite bundle is cut transversally and consists of several groups of neurites, which are accompanied by different types of perikarya (Figure 10A). There are three types of neurites: those with dense-core synaptic vesicles, clear synaptic vesicles, and medium-dense synaptic vesicles. Synaptic contacts occur between the neurites (Figure 10B). The minor nerve ring is also formed by several bundles, most of which contain synaptic vesicles with medium-dense content (Figure 10C). In sagittal sections of larvae, most neurites of the minor nerve ring are oriented longitudinally and form a large mediofrontal bundle that extends into each tentacle.

**Figure 11:**
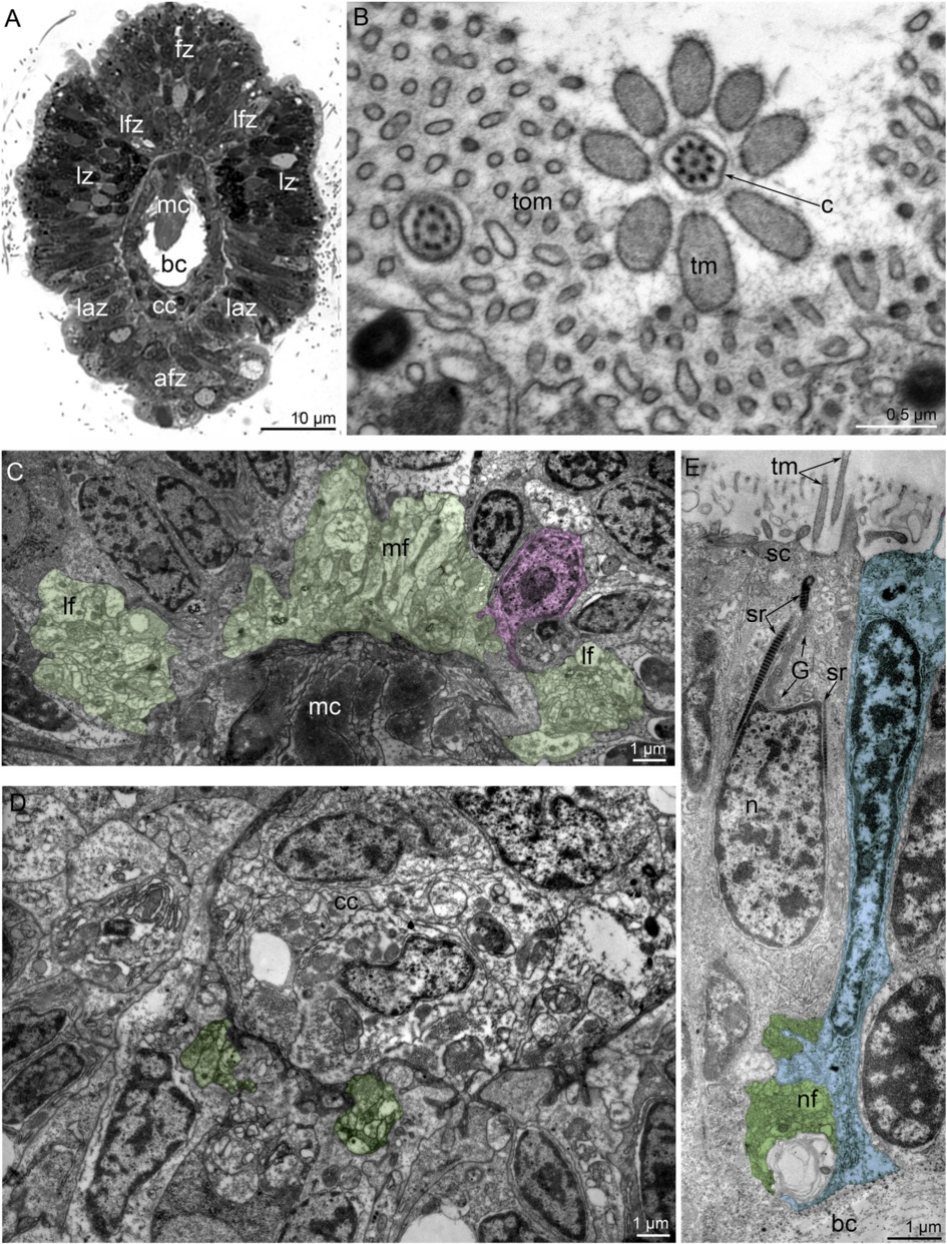
**Organization of tentacles in competent larvae of *Phoronopsis harmeri*.** Semi-thin cross section **(A)** and thin cross sections **(B-E). (A)** Section of the middle part of a tentacle. Around each tentacle, there are several zones of epidermis: frontal (fz), lateral (lz), abfrontal (afz), two laterofrontal (lfz), and two lateroabfrontal (laz). **(B)** Section of the apical portion of the laterofrontal sensory cell with a cilium (c) and thick microvilli (tm), which are much thicker than regular microvilli (tom). **(C)** The mediofrontal (at the top) and two laterofrontal (to the sides) neurite bundles (green). A nonsensory perikaryon (pink) is associated with the mediofrontal neurite bundle. **(D)** Two small lateroabfrontal neurite bundles (green). **(E)** The portion of the laterofrontal epidermis with the sensory laterofrontal cell (sc), which bears thick microvilli, and an additional sensory cell (blue) that contacts the laterofrontal neurite bundle (green). Abbreviations: afz – abfrontal zone of tentacle; bc – blastocoel; c – cilium; cc – cells of coelomic lining; G – Golgi apparatus; laz – lateroabfrontal zone of tentacle; lf – laterofrontal neurite bundle; lfz – laterofrontal zone of tentacle; lz – lateral zone of tentacle; mc – muscle cells; mf – mediofrontal neurite bundle; n – nucleus; nf – nerve fibers; sc – laterofrontal sensory cell; sr – striated rootlet; tm – thick microvilli, tom – thin microvilli.

In competent larvae, each tentacle has several zones, which differ in the organization of the epidermis (Figure 11A). Each tentacle contains five neurite bundles. The largest bundle is mediofrontal. It consists of 80-100 neurites and is located strictly above the tentacle elevator (Figure 11C). The mediofrontal neurite bundle is closely associated with perikarya, which contain a few clear synaptic vesicles and nuclei with electron-lucent karyoplasm and large nucleoli (Figure 11C). Two laterofrontal neurite bundles are associated with the sensory laterofrontal cells. Each laterofrontal bundle consists of 40-50 neurites (Figure 11C). Sensory cells are arranged along each tentacle in two rows. Each of these cells has a cilium, which is surrounded by eight, thick microvilli (Figure 11B). The basal body of the cilium gives rise to three striated rootlets, which pass along the nucleus (Figure 11E). Common epidermal cells, which are also apparently sensory, occur near typical sensory cells with thick microvilli (Figure 11E). The basal parts of these epidermal cells form projections, which contain synaptic vesicles. Paired lateroabfrontal neurite bundles are usually associated with gland cells. These are the smallest bundles and consist of 15-20 neurites (Figure 11D).

The midgut contains many cells, which have an unusual organization relative to that of the regular epithelial cells (Figure 10D). Each of these unusual cells contains a large, roundish nucleus with electron-lucent karyoplasm and a large nucleolus. The cell cytoplasm is filled with numerous electron-dense granules, with diameters ranging from 100 to 115 nm. The same granules were found in neurites, which are visible in the basal portion of the midgut epithelium (Figure 10D).

In the epidermis of the oral field, small bundles of neurites and perikarya are evident with TEM. The perikarya contain dense-core vesicles. The neurites are filled with large, dense-core vesicles and vesicles with medium-dense content (Figure 10E).

The telotroch nerve ring is located in the epidermis between the trunk wall and telotroch (Figure 10F). It consists of 5-7 neurites of different diameters. Large neurites have light cytoplasm and contain clear synaptic vesicles. Small neurites contain dense-core vesicles.

### Organization of the nervous system in competent larvae

Taken together, our results reveal that the nervous system in competent larvae of *P. harmeri* consists of the following elements: an apical organ, a median neurite bundle, an anterior and posterior marginal neurite bundle, a frontal organ and a sensory field, a tentacular neurite bundle (main nerve ring), two dorsolateral groups of perikarya, a minor nerve ring, five radial neurite bundles in each tentacle, a telotroch nerve ring, an anal nerve ring, neurites and perikarya in the epidermis of the oral field, the trunk, an esophagus, a metasomal sac, and a midgut (Figure 4B).

## Discussion

### Changes of phoronid nervous system anatomy during larval development

The nervous systems of phoronid larvae have been well studied by a variety of methods, including light microscopy [33], TEM [14,28,30], immunocytochemistry [27,29], and confocal laser scanning microscopy [16,18,19,31]. Previous studies usually involved precompetent larvae, but the nervous system of phoronid larvae changes greatly before metamorphosis. According to our results, these changes mostly concern the preoral lobe of the larva, which apparently plays a main role in larval settlement [4]. The organization of the median neurite bundle becomes very complicated with age. At all larval stages, it consists of two lateral and one median bundles, but only lateral bundles exhibit serotonin-like immunoreactivity in precompetent larvae. Interestingly, although the median bundle in competent larvae exhibits serotonin-like immunoreactivity, the distal end of this bundle does not display this activity. At the same time, the distal portion of the median bundle exhibits FMRFamide-like immunoreactivity. The frontal organ appears in the distal portion of the median neurite bundle. This is the second nerve center, which appears before metamorphosis in all phoronid larvae [2]. In some phoronid larvae, the frontal organ sticks out from the epidermis surface, whereas in other larvae, it is not everted above the epidermis surface and is not visible in live larvae [16]. In competent larvae of *P. harmeri*, the frontal organ is not everted and can be recognized in sections. The serotonin-like immunoreactive part of the frontal organ includes two groups of perikarya: frontal and median. The aggregation of perikarya near the edge of the midline of the hood was found in some competent phoronid larvae [16], whereas the frontal perikarya, which form a huge group (a sensory field), are described for the first time in the current report. In young larvae of *P. muelleri*, several serotonin-like immunoreactive monopolar perikarya occur along the median part of the epistome edge, but their connection with median and marginal neurite bundles is unclear [27].

Perikarya of the sensory field are associated with the anterior neurite bundle, which exhibits serotonin-like immunoreactivity in young [19] and in competent (herein) larvae of *P. harmeri* but not in precompetent larvae of *P. harmeri*. The plasticity of the timing when some nerve elements repeatedly appear and disappear during phoronid development was previously mentioned [18,19,29]. This plasticity also relates to the distribution of neurites and perikarya of the oral field of the larvae. Interestingly, the oral field, which is well innervated in young larvae of *P. harmeri*[18,19], is nearly without immunoreactive perikarya and neurites in competent larvae. Competent larvae have a single nerve ring of the telotroch, whereas the second (posterior) nerve ring, which is evident in young larvae [19], cannot be found by immunocytochemistry or TEM.

The other changes of the larval nervous system concern the increase in the number of perikarya in the apical organ, in the midgut, and in the epidermis of the trunk.

In summary, we conclude that the most prominent event in development of the larval nervous system correlates with formation of the frontal organ. In competent larvae, the most important structure is the frontal organ, which is used to select the substratum for settlement. The video file indicates that the larva uses the frontal organ to scan the substratum (in the video, the substratum is a piece of tube of an adult *P. harmeri*). During the scanning, the shape of the preoral lobe changes greatly (Additional file 1). At the same time, phoronids do not use the frontal organ to attach to the substratum in the manner of bryozoans. In bryozoans, the frontal organ contains numerous gland cells, which produce a sticky secretion that helps the larvae attach to the substratum [34].

### Nervous system of phoronid larvae

Among phoronid larvae, two types of nervous system organization have been recognized [16]. According to Santagata and Zimmer, “**Type 1** species have two fiber-rich, dorsal serotonergic nerves that extend from the apical ganglion and are continuous with the main nerve ring. This type also has two lateral serotonergic processes that form part of the minor nerve ring and the abfrontal nerves of the larval tentacles. Catecholaminergic fibers from the main hood nerve are continuous with the frontal larval tentacle nerves. **Type 2** species have two fiber-poor, dorsal serotonergic nerves that extend from the apical ganglion and are continuous with the main nerve ring. These species exhibit a fiber-rich, serotonergic, and catecholaminergic main hood nerve from which all fibers of the minor nerve ring and larval tentacle nerves originate” [16].

The organization of the nervous system of *P. harmeri* larvae, however, does not fit either type. It differs from type 1 in its lack of the “lateral serotonergic processes”. These paired processes extend from the apical organ and contact the minor nerve ring, which gives rise to the abfrontal nerves of the tentacles [16]. The nervous system of *P. harmeri* larvae is more similar to the type 2 than type 1 nervous system, but differs from the type 2 in the innervation of the tentacles. In larvae with the type 2 nervous system, all tentacular nerves originate from the minor nerve ring, whereas only the mediofrontal tentacular nerves originate from the minor nerve ring in *P. harmeri* larvae.

The innervation of the tentacles in phoronid larvae is controversial. According to Hay-Schmidt’s immunocytochemistry data [27], young larvae of *P. muelleri* have six neurite bundles in each tentacle. One mediofrontal neurite bundle consists only of catecholamine-containing neurites. Two laterofrontal neurite bundles exhibit catecholamine- and FMRFamide-like immunoreactivity. Two lateroabfrontal neurite bundles consist of catecholamine- and serotonin-like immunoreactive neurites. One medioabfrontal neurite bundle exhibits serotonin-like immunoreactivity. The medioabfrontal neurite bundle originates from two groups of “lateral epistome-mesosome processes”, which extend from the neuropile towards the ventrolateral side of the oral field where they branch once or twice before one branch extends into the tentacle. These branches do not skirt the tentacles (see Figure 3A,B in [27]), and their location on the abfrontal side of tentacle is unclear, because the epidermis of the oral field continues to the frontal but not to the abfrontal side of the tentacle. Because the neurites are intraepidermal, they must follow the epidermis; for innervation of the abfrontal side of tentacles, neurites of the oral field must skirt the tentacle bases. At the same time, according to Hay-Schmidt’s ultrastructural data [28], larva of *P. muelleri* has one mediofrontal, two laterofrontal, and two small lateroabfrontal neurite bundles in each tentacle.

On the other hand, according to Santagata and Zimmer [16], some phoronid larvae (those with the type 1 nervous system) lack neurites along the frontal side of tentacles. Moreover, these authors suggested that the lateroabfrontal neurites originate from the minor nerve ring, whereas our observations and those of Hay-Schmidt [27] indicate that these neurites originate from the main nerve ring.

The question of how the tentacles are innervated in phoronid larvae requires additional investigation because comparative analysis of tentacle innervation is important for distinguishing among groups of “lophophorates”. Our results indicate that the innervation of the larval tentacles involves both the main and minor nerve ring.

In general, the serotonin-like immunoreactive nervous system is more complicated in competent larvae of *P. harmeri* than in other phoronid larvae studied to date. First, competent larvae of *P. harmeri* have two serotonin-like immunoreactive marginal neurite bundles (the anterior bundle, which continues to the oral field, and the posterior bundle, which is associated with serotonin-like immunoreactive cells of two types and gives rise to the minor nerve ring); other phoronid larvae have only one marginal neurite bundle (the posterior bundle) [16,27]. Second, competent larvae of *P. harmeri* have a “sensory field” on the edge of the hood midline. Third, competent larvae of *P. harmeri* have numerous neurites and perikarya along the larval trunk and the telotroch nerve ring. These nerve elements have been observed in *P. muelleri* larvae [27] but not in other phoronid larvae studied to date [16,30]. Fourth, competent larvae of *P. harmeri* have perikarya that are scattered along the main nerve ring and form two dorsolateral groups in the sites of the youngest tentacles. Fifth, among all phoronid larvae studied to date, the innervation of inner organs has been observed only in *P. harmeri*. The innervation of the esophagus and cardiac sphincter in *P. harmeri* larvae is provided by both serotonin-like and FMRFamide-like immunoreactive neurites [35], herein. The small number of FMRFamide-like immunoreactive perikarya that occur in the midgut of young larvae of *P. harmeri*[35], increase to as many as 30 in competent larvae.

### Ultrastructure

Our ultrastructural data provide new information about the organization of the nervous system in phoronid larvae. In general, these data corroborate the results of Hay-Schmidt [28], Lacalli [30], and Santagata [31]. The apical organ consists of several types of perikarya. According to the location of the perikarya of the apical organ, we can infer that they belong to the serotonin-like immunoreactive sensory cells (perikarya of the first type), serotonin-like immunoreactive cells under the neuropil (perikarya of the second type), and FMRFamide-like immunoreactive non-sensory cells (perikarya of the third type). According to Santagata’s data [31], the organization of the apical organ is simpler for competent larvae of *P. pallida* than for *P. muelleri*[28] or *P. harmeri*[19], herein competent larvae. Thus, the apical organ of *P. pallida* lacks non-sensory serototin-like immunoreactive cells, which are located under the neuropil in the apical organs of *P. muelleri* and *P. harmeri*. Interestingly, the apical organ of young larvae of *P. vancouverensis* also lack perikarya under the neuropil [30]. The differences in the organization of the apical organ can be explained by differences in larval biology: *P. pallida* and *P. vancouverensis* larvae live in plankton for only 1 month, whereas *P. harmeri* larvae live in plankton for 3 months.

The organization of the frontal organ seems similar in competent larvae of both *P. harmeri* and *P. pallida*[31]. In *P. pallida* larvae, the frontal organ has a large neuropil, which is located close to the apical organ but does not contain any serotonin- or catecholamine-like immunoreactive perikarya. At the same time, TEM reveals sensory and nonsensory cells around the neuropil of the frontal organ of *P. pallida*[31]. In *P. harmeri* larvae, the frontal organ is distant from the apical organ and contains several types of perikarya, which were revealed with both TEM and immunocytochemical methods.

The organization of the nervous system of the larval hood differs among the studied species. Thus, larvae of three species (*P. harmeri*, *P. muelleri*, and *P. vancouverensis*) have both anterior and posterior marginal neurite bundles, whereas actinotrocha C, actinotrocha D, and larvae of *P. harmeri* from the Pacific Coast of North America have only a single marginal neurite bundle [16]. The marginal neurite bundles in *P. muelleri* larvae do not exhibit serotonin-like immunoreactivity as they do in *P. harmeri* larvae. At the same time, the posterior marginal neurite bundle is located very distant from the hood edge in *P. muelleri* larvae [28] but is located much closer to the hood edge in larvae of *P. vancouverensis*[30] and *P. harmeri* [herein]. In young larvae of *P. vancouverensis*[30], “glial-like capsular” cells are associated with the posterior marginal neurite bundle.

TEM revealed that the innervation of tentacles is more complicated in *P. harmeri* larvae than in other phoronid larvae [28,30]. Besides the longitudinal neurite bundles, which occur in the tentacles of other phoronid larvae, *P. harmeri* larvae have additional sensory cells that accompany the laterofrontal sensory cells and the non-sensory perikarya associated with the mediofrontal neurite bundle. The ultrastructural organization of the laterofrontal sensory cells in the tentacles of *P. harmeri* larvae seems very similar to that for adult phoronids [36].

The oral field is well innervated in young phoronid larvae [18,19,27,29,30]. At the same time, in competent larvae, the neurites and perikarya of the oral field do not exhibit immunoreactivity [16,31], herein and can be detected only by TEM [herein].

In *P. harmeri* larvae, the main tentacular neurite bundle contains many perikarya of different types, and synaptic contacts occur between the neurites. Perikarya have never been found in the tentacular neurite bundle of other phoronid larvae [16,27-31], perhaps because the studied larvae were noncompetent [27-30] and the nervous system organization of noncompetent larvae is likely to be simple. This difference in the organization of the tentacular neurite bundle may reflect the general difference in the organization of the whole nervous system among different phoronid larvae.

The innervation of the internal organs was described previously [35], and our recent data are consistent with these earlier findings. The ultrastructure of perikarya in the midgut seems very similar in both *P. harmeri* larvae and actinotrocha sp. [35]. These cells lack synaptic vesicles and are filled with electron-dense granules, whose presence is a hallmark of neurosecretory cells [37]. Neurosecretory cells are common in the digestive epithelium, where they control the movement of food.

### Organization of the nervous system in Brachiozoa

As mentioned earlier, phoronids are regarded as the closest relatives of brachiopods [8-10]. Phoronids and brachiopods exhibit at least two types of development: planktotrophic and lecithotrophic. The nervous system is very complex (consists of many different elements) for planktotrophic larvae [16,38] but is usually very simple for lecithotrophic larvae and consists only of an apical organ with a small number of cells and a pair of longitudinal neurite bundles [26,39]. Two important questions arise: Which type of development arose first in the Brachiozoa: planktotrochic or lecithotrophic? Which type of nervous system is plesiomorphic among the Brachiozoa? According to paleontological data [40], planktotrophy may be regarded as the primary condition of brachiopods. For this reason, we suggest that the larvae of the last common brachiozoan ancestor had tentacles that were used to feed. Therefore, the presence of planktotrochic larvae with nervous systems consisting of many elements may be regarded as the primary condition of the Brachiozoa.

### Serotonin-like immunoreactive nervous system in bilaterian ciliated larvae

Traditionally, the development and organization of the nervous system are among the most important characteristics for the reconstruction of animal phylogeny. Two main groups of Bilateria (the deuterostomes and protostomes) differ in the organization of the serotonin-like immunoreactive nervous system [11]. This difference especially concerns the organization of the apical organ: the apical organ contains numerous serotonin-like immunoreactive cell bodies (some of which form two clusters connected by a commissure) in deuterostomian larvae but few serotonin-like immunoreactive cell bodies in protostomian larvae. In competent as well as in young phoronid larvae [19], the apical organ includes two clusters of nonciliate serotonin-like cell bodies, which give rise to the two branches of the tentacular neurite bundle. This prominent neurite bundle passes along the postoral ciliated band in phoronid larvae. The presence of bilateral symmetrical apical organ, which gives rise to the prominent neurite bandle that passes along the postoral ciliated band, is known in many deuterostomian larvae [41-44]. In contrast, most protostomian larvae lack nerve elements that conduct the postoral ciliated band. Moreover, in spiralian trochophores, the apical organ never directly connects to the neurite bundles that innervate the ciliated bands [45-48]. In these ways, the serotonin-like immunoreactive nervous system of competent larvae of *P. harmeri* has more in common with those of deuterostomian than protostomian larvae (Table 1). At the same time, the development of the *P. harmeri* nervous system combines protostomian and deuterostomian features [18,19].

**Table 1:** Features of the organization of the serotonin-like immunoreactive nervous system in ciliated larvae of phoronids and in some protostomian and deuterostomian organisms

**Feature**	**Phoronida**	**Protostomia**	**Deuterostomia**
		**Nemertea, Polychaeta, Mollusks**	**Hemichordata, Echinodermata**
	[16,18,19,27-31]** herein**	[45-52]	[41-44,53]
location where the first perikarya appear	always on the anterior pole of the embryo	mostly on the anterior pole, but on the posterior pole of the embryo in some species	always on the anterior pole of the embryo
location where the first neurites appear	neurites under the postoral ciliated band	neurites under the preoral ciliated band	neurites under the postoral ciliated band
number of perikarya in the apical organ	many	few	many
	(more than 50)	(2-12 perikarya)	(more than 30)
organization of the neurite bundle underlying the postoral ciliated band:			
1) present/absent	1) +	1)― (in the most of protostomia larvae) + [46]	1) +
2) presence of the connection with apical organ	2) +	2) ― (even if neurite bundle exists)	2) +
3) presence of perikarya among neurites	3) + [herein] ― [28,30,31]	3) ― (even if neurite bundle exists)	3) +
presence of the telotroch nerve ring	+	―	+
presence of the ventral nerve cord in early development	+	+	―

“ + ” indicates the presence of the feature; “―” indicates its absence.

### The nervous system in bilaterian ciliated larvae and the ground plan of the bilaterian nervous system

There is a classic controversy in zoology about whether the common ancestor of living bilateria was a benthic animal with a bilaterian body plan or a pelagic larva-like animal similar to what we see today in the primary larvae of indirect-developing bilaterians (for review, see [54,55]). Although most recent authors believe that the feeding larvae are specializations of the ontogeny of an ancestral, direct development [56-58], the organization of ciliated larvae is still useful for evolutionary and phylogenetic deductions [20,21], especially with respect to the development and organization of the nervous and muscular systems. According to modern analysis [59-61], the last common bilaterally symmetrical ancestor had a centralized nervous system, which consisted of a nerve center and nerve cords that innervated the tentacles. The nerve center had a simple histological organization, consisting of perikarya and a neuropil, and did not contain any additional (glial) cells [60]. This organization is evident in the larvae of many extant bilaterian groups. The organization of the nerve center of the last bilaterian ancestor may have consisted of many or only a few cells. Nerve centers with many cells occur in larvae of the following extant bilaterian: phoronids [19], deuterostomians [41,42], some mollusks [45], and entoprocts [21]. Whereas those bilaterians having nerve centers consisting of many cells belong to two different stems of Bilateria (Protostomia and Deuterostomia), those with nerve centers consisting of only a few cells occur only in the Protostomia. For this reason, it seems logical to infer that the last common bilaterian ancestor had a nerve center consisting of many cells and that the cell number was reduced in some groups as a result of lecithotrophy (as in some brachiopods [26]) or determination of early development (as in Spiralia).

Based on all of these data, we suggest that phoronid larvae have retained some features of the ancestral nervous system and can be regarded as more primitive than their spiralian “relatives”. It follows that phoronids should be extracted from the Trochozoan (=Spiralia) clade and placed at the base of the Lophotrochozoan stem. This idea was first suggested by Peterson and Eernisse [62] and has been confirmed by other results [1,5,14,63].

## Conclusion

The organization of the nervous system is more complicated in the competent larvae of *P. harmeri* than in any other phoronid larvae studied to date. Before metamorphosis, the nervous system *P. harmeri* larvae includes the bilaterally symmetrical apical organ, the tripartite median neurite bundle with frontal organ and a sensory field on the distal end, the anterior and posterior marginal neurite bundles, two dorsolateral groups of perikarya, main and minor nerve rings, five radial neurite bundles in each tentacle, trunk neurites and perikarya, the telotroch nerve ring, and perikarya and neurites of the esophagus, the midgut, and the metasomal sac. The organization of the nervous system differs among phoronid larvae, and these difference reflect the presence of different types of phoronid planktotrophic larvae [2,16]. Comparative analysis of the nervous system organization in lophotrochozoan and deuterostomian larvae revealed that the overall anatomy of the nervous system of phoronid larvae has more in common with the deuterostomian pattern than with protostomian pattern. The most important distinguishing characteristic is that phoronid and deuterostomian larvae but not protostomian larvae have a bilaterally symmetrical apical organ that contains many serotonin-like immunoreactive perikarya and that gives rise to the prominent neurite bundle passing along the postoral ciliated band. At the same time, the presence of the apical organ and the nerve cords, which innervate the tentacles, is the most plesiomorphic condition of the bilaterian nervous system. For this reason, phoronids can be regarded as primitive lophotrochozoans.

## Methods

### Animals

Advanced and competent larvae of *P. harmeri* were collected with a planktonic net during November of 2011 in Vostok Bay, Sea of Japan (for details see [64]. Larvae were reared at 1 to 3°C in an incubator with a 12-h light–dark cycle until metamorphosis.

Advanced and competent larvae were photographed using a Panasonic DMC-TZ10 digital camera mounted on a binocular light microscope. All larval stages were prepared for scanning electron microscopy (SEM), transmission electron microscopy (TEM), cytochemistry, and confocal laserscanning microscopy (CLSM).

For SEM, fixed larvae of *P. harmeri* that had been dehydrated in ethanol followed by an acetone series were critical point dried and then sputter coated with platinum-palladium alloy. Specimens were examined with a Jeol JSM scanning electron microscope.

For TEM, larvae of *P. harmeri* were fixed at 4°C in 2.5% glutaraldehyde in 0.05 M cacodylate buffer containing 21 mg/ml NaCl and then postfixed in 2% osmium tetroxide in the same buffer containing 23 mg/ml NaCl. Postfixation was followed by *en bloc* staining for 2 h in a 1% solution of uranyl acetate in distilled water. Specimens were then dehydrated in ethanol followed by an acetone series and embedded in Spurr resin (Sigma Aldrich). Semi-thin and thin sections were cut with a Reichert Ultracut E ultratome. Semi-thin sections were stained with methylene blue, observed with Zeiss Axioplan2 microscope and photographed with an AxioCam HRm camera. Thin sections were stained with lead citrate and then examined with a JEOL JEM 100B electron microscope.

For cytochemistry, advanced and competent larvae of *P. harmeri* were narcotised in MgCl2, then fixed overnight in a 4% paraformaldehyde solution on a filtrate of sea water and washed (two times) in phosphatic buffer (pH 7.4) (Fisher Scientific) with Triton X-100 (0.3%) (Fisher Scientific, Pittsburgh, PA, USA) for a total of 2 h. Nonspecific binding sites were blocked with 1% normal donkey serum (Jackson ImmunoResearch, Newmarket, Suffolk, UK) in PBT overnight at +4°C. Subsequently, the larvae were transferred into primary antibody: the mixture of a-Acetylated Tubulin (1:1000) and either anti-FMRFamide (1:3000) or anti-serotonin (1:1000) (ImmunoStar, Hudson, WI, USA) in PBT and incubated for 24 h at +4°C with gentle rotation. Specimens were washed for 8 h at +4°C (at least three times) in PBT and then exposed to the secondary antibody: donkey anti-rabbit- Atto 647 N and donkey anti-mouse-Atto 565 (Invitrogen, Grand Island, NY, USA) both 2-3 mkg/ml in PBT for 24 h at +4°C with gentle rotation. In the following, they were washed in PBS (three times × 40 min), mounted on a cover glass covered with poly-L-lysine (Sigma-Aldrich, St. Louis, MO, USA), and embedded in Murray Clear. Specimens were viewed with Leica TCS SP5 confocal microscope (IDB, Moscow, Russia). Z-projections were generated using the programme Image J version 1.43. Three-dimensional reconstructions were generated using Amira version 5.2.2 software (Bitplane, Zurich, Switzerland).

### Terminology

In this report, we have used the terms suggested by Russel Zimmer [33], who used light microscopy to provide the first complete description of the nervous system of phoronid larvae. We have also used some terms suggested by Scott Santagata [16,31] and specific neuroscience terms proposed by Richter and colleagues [65].

## Competing interest

The authors declare that they have no competing interests.

## Authors’ contributions

ENT designed and coordinated research, performed research including staining and confocal research, TEM investigations, analyzed data, prepared all figures, and wrote the manuscript. EBT performed staining and confocal research. Both authors conceived the study, read, and approved the final version of the manuscript.

## Supplementary Material

Additional file 1: Movie 1 The competent larva of *Phoronopsis harmeri*. This time-lapse movie shows the typical behavior of competent larvae before settlement. The larva used a piece of the tube of an adult *P. harmeri* as a substratum for metamorphosis. In the movie, the piece of the tube is the white stone to the left. The larval hood always moves and touches the piece of tube. At this moment, the hood elongates along midline, and then the larva begins to metamorphose. Click here for file
